# Advances in the Study of Gas Hydrates by Dielectric Spectroscopy

**DOI:** 10.3390/molecules26154459

**Published:** 2021-07-24

**Authors:** Ivan Lunev, Bulat Kamaliev, Valery Shtyrlin, Yuri Gusev, Airat Kiiamov, Yulia Zaripova, Artur Galiullin, Abdolreza Farhadian, Mikhail Varfolomeev, Malcolm Kelland

**Affiliations:** 1 Department of Radio Electronics, Kazan Federal University, 18 Kremlevskaya Street, 420008 Kazan, Russia; bulat1596@gmail.com (B.K.); ygusev@mail.ru (Y.G.); argj95@gmail.com (A.G.); 2 Department of Inorganic Chemistry, Kazan Federal University, 18 Kremlevskaya Street, 420008 Kazan, Russia; valery.shtyrlin@gmail.com; 3 Department of General Physics, Kazan Federal University, 18 Kremlevskaya Street, 420008 Kazan, Russia; airatphd@gmail.com; 4 Department of Physical Chemistry, Kazan Federal University, 18 Kremlevskaya Street, 420008 Kazan, Russia; yu-ya98@yandex.ru; 5 Department of Petroleum Engineering, Kazan Federal University, 18 Kremlevskaya Street, 420008 Kazan, Russia; AFarhadian@kpfu.ru; 6 Department of Chemistry, Bioscience and Environmental Engineering, Faculty of Science and Technology, University of Stavanger, N-4036 Stavanger, Norway; malcolm.kelland@uis.no

**Keywords:** dielectric spectroscopy, gas hydrates, kinetic hydrate inhibitors, dielectric relaxation

## Abstract

The influence of kinetic hydrate inhibitors on the process of natural gas hydrate nucleation was studied using the method of dielectric spectroscopy. The processes of gas hydrate formation and decomposition were monitored using the temperature dependence of the real component of the dielectric constant ε′(T). Analysis of the relaxation times τ and activation energy ΔE of the dielectric relaxation process revealed the inhibitor was involved in hydrogen bonding and the disruption of the local structures of water molecules.

## 1. Introduction

Since the first report [1] that gas hydrates were responsible for blocking pipelines, they were considered to be a serious problem in the production and transportation of oil and gas. Gas hydrates are formed at a high pressure and low temperature and are composed of gas molecules such as methane, ethane or propane which are trapped in the hydrogen-bonded framework of water molecules [2]. In addition, hydrate formation can occur in porous media [3,4], which significantly affects the efficiency of field development. Therefore, studying the natural gas hydrate formation process in terms of the thermodynamics and kinetics of phase transitions is a relevant fundamental and applied issue. Increasing the efficiency and the safety of hydrocarbon production and fluid transportation, and developing technologies for the extraction of methane from natural gas hydrate deposits, are urgent issues that need to be resolved. Another important issue relating to the fundamental aspects of investigating hydrate formation is the identification of a hydrate formation mechanism and its structural type. Several experimental apparatuses have been developed to study hydrate formation and rank the effectiveness of hydrate inhibitors over the years [5,6]. The most widely reported instruments include differential scanning calorimeters, stirred reactors, automated lag time apparatuses (HP-ALTA), rocking cells and flow loops [7,8]. The selection of the proper experimental technique is an important factor in determining the data quality for a specific research goal. It has been reported [9,10,11] that dielectric spectroscopy is a powerful method for studying emulsions and other complex systems. However, the phase state (ice and/or inclusion compounds) and the nature and dynamics of phase transitions in systems containing hydrate forming agents have only been studied using this method in a few works [12,13,14]. In addition, a dielectric measuring cell has been used to artificially produce gas hydrates [15]. In the present work, we propose the design of a new dielectric cell allowing one to study the formation/decomposition of gas hydrates under pressure. It was found that the dielectric properties of gas hydrates are significantly different from those of hexagonal ice, with kinetic hydrate inhibitors influencing these properties. This makes it possible to further study the effects of various reagents on the kinetics of gas hydrate formation/decomposition using dielectric spectroscopy.

## 2. Results and Discussion

**Figure 1:**
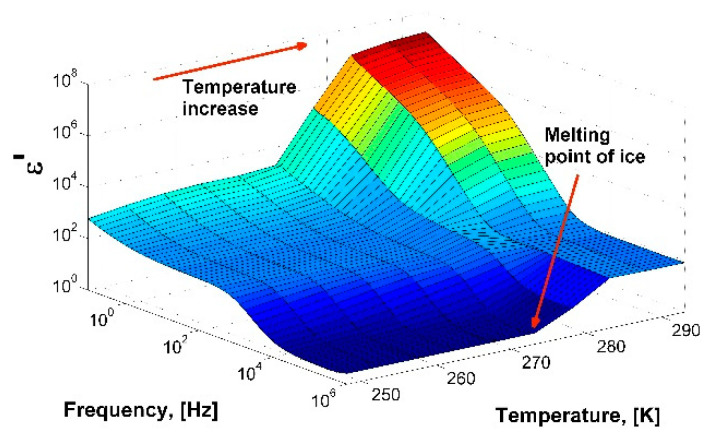
A 3D-diagram of the real part of dielectric spectrum of ice/water in the temperature range from 253 to 293 K.

To check the operability of the measuring cell, distilled water with a volume of 1.3 cm^3^ was poured into the cell and the dielectric spectra were measured with temperatures ranging from 253 to 293 K. The sample was cooled to a temperature of 253 K at a rate of 5 K/min, then heated to a temperature of 293 K at a rate of 3 K/min. Figure 1 shows a three-dimensional diagram of the real part of the dielectric spectrum of ice (below 273 K) and water in the temperature range from 253 to 293 K. The calculated dependences τ (1000/T) and ε_s_ (T) agree well with the data obtained earlier [16,17,18,19], which confirms the efficiency of the constructed measuring cell (Figure S1; See Supplementary Meterials).

The obtained dependence τ (1000/T) is in good agreement with the data produce by Johari et al. [16] in the high-temperature range 240–273 K. At lower temperatures, there is a discrepancy in the relaxation times; they have shorter values. The reason for the discrepancy is that the dielectric response of ice I*h* strongly depends on its preparation and the temperature measurement protocol, especially at low temperatures. The microstructure of ice affects the migration of ionic and Bjerrum L-D defects [17] and the relationship between them. This property determines the dynamics at low temperatures, where there is a discrepancy between the results. At high temperatures, the mobility of defects is high, so any deviations in ice preparation are averaged. Therefore, above T ≈ 240 K, measurements give approximately the same values of τ. A detailed comparison of the experimental data for ice I*h* and an analysis of the different behaviors of the dynamic parameters at low temperatures are described in detail in the work [20].

**Figure 2:**
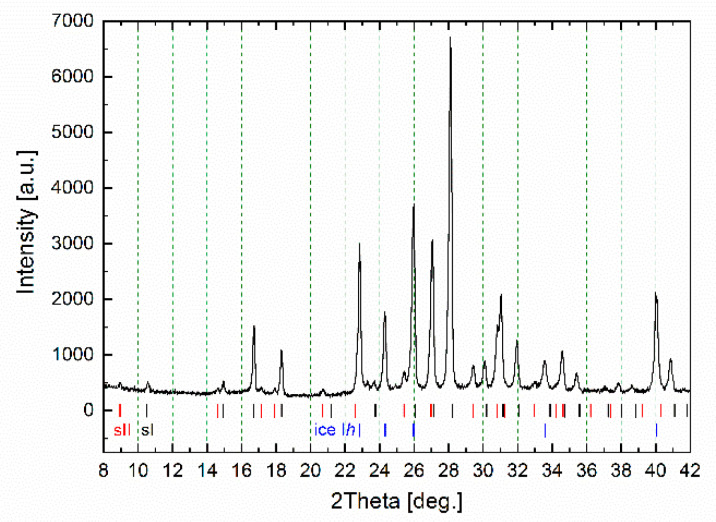
Powder X-ray diffraction pattern of the studied gas hydrate measured at 173 K; sI, sII and ice I*h* correspond to the positions of reflections of the cubic structure I, cubic structure II hydrates and hexagonal ice.

The hydrate formation/decomposition process was studied in a self-developed dielectric cell (see Section 3. Materials and Methods) with a synthetic natural gas (the molar percentage composition was 95.7% CH_4_, 2.6% C_2_H_6_, 0.8% C_3_H_8_, 0.1% C_4_H_10_, 0.1% i-C_4_H_10_, 0.7% N_2_) as the hydrate-forming gas. The calculated equilibrium conditions of the formation of sII gas hydrate from the synthetic natural gas used are shown in Figure S2 (Supplementary). One of the obtained samples was frozen to liquid nitrogen temperature and the quenched sample was taken out to be studied using powder X-ray diffraction (Figure 2). Analysis of the diffraction patterns was carried out on the basis of literature data [21,22]. The hydrates with cubic structure I (sI) and cubic structure II (sII) as well as hexagonal ice I*h* were revealed in the prepared sample. As the hydrate formation usually takes place at the water–gas interface, it should be mixtures of sI + sII and sI + sII + ice I*h* phases which occurred in the dielectric spectroscopy measurements above and below ice freezing point respectively.

In the case of dielectric spectroscopy measurements, the measuring condenser (total volume 1.3 mL) was filled with 0.8 mL of distilled water, leaving a remaining 0.5 mL for water-gas contact. The conditions for obtaining the hydrate (273 K, 9 MPa) were selected experimentally and allowed the hydrate to be obtained in a relatively short time period while avoiding freezing. Based on the P, T section of the phase diagram (Figure S2; Supplementary), the equilibrium temperature of hydrate formation at 9 MPa is 289.5 K, resulting in subcooling equal to 16.5 K under experimental conditions.

**Figure 3:**
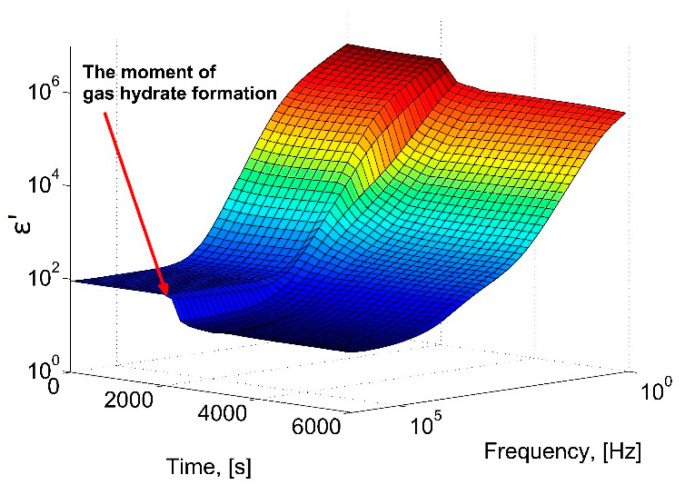
A 3D-diagram of the dependence of the real part of the dielectric constant during the hydrate formation at 273 K, 9 MPa.

Figure 3 shows a three-dimensional diagram of the change in the real part of the dielectric constant of the water-gas system at a constant gas pressure of 9 MPa, depending on the time and frequency of the electric field. After a period of about 33 min (2000 s), a sharp decrease in the ε′ amplitude occurs, which corresponds to the beginning of the formation of the gas hydrate.

**Figure 4:**
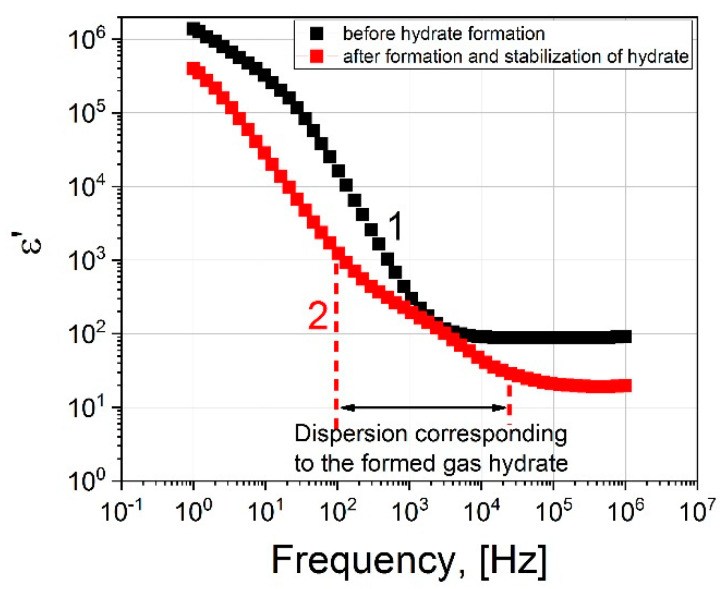
The real part of the dielectric spectrum: black squares correspond to the spectrum before the hydrate formation; red squares represent the spectrum after hydrate formation at 273 K, 9 MPa.

Figure 4 shows the real parts of the dielectric spectrum before the formation of the hydrate (1) and after (2).

**Figure 5:**
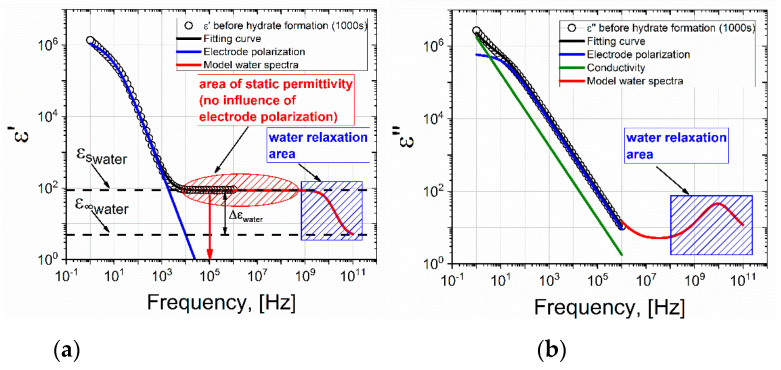
(**a**) Real and (**b**) imaginary parts of the dielectric spectrum of the water-gas system at the time t = 2000 s, before the formation of the hydrate (273 K, 9 MPa). The black line represents the fitting function constructed in accordance with equation (2); the blue line represents the contribution of electrode polarization; the green line is DC conductivity; and the red line is the model spectrum of water.

Let us consider in detail the dielectric spectrum (1) upon reaching the time t = 2000 s. Figure 5 shows the real and imaginary parts of the dielectric spectrum.

Electrode polarization (EP) was observed at a low frequency and a plateau was observed in the frequency range 10^4^–10^6^ Hz, which corresponded to the level of static permittivity of water ε_s_. It should be noted that the EP is typically either not taken into account at all during data processing, or is described by the Jonscher correction [23]. However, the Jonscher equation ∼(iω)^−n^ is a simple power law of behavior and in many cases does not correspond to experimental data. Indeed, very often bends and/or humps [24,25,26,27] are found at the low-frequency end of the EP, which cannot be described by the Jonscher correction. There are many theoretical models that improve the description of EP [28,29], but here we will use the results of our previous studies [30,31], where this issue was considered in detail. In these studies, it was shown that the EP tail should be described by the following frequency-dependent term

(1) $$\varepsilon_{EP}(\omega )=\frac{\Delta \sigma }{i\omega \varepsilon_{0}\left(1+{\left(i\omega \tau_{EP}\right)}^{-\alpha_{EP}}\right)},$$

where Δσ is the drop in conductivity due to the appearance of a double electric layer; τ_EP_ corresponds to the relaxation time of the formation of a double layer; and α_EP_ characterizes the dynamics of diffusion of charge carriers near the electrode surfaces [30,32]. Expression (1) correctly describes any bends in the tail of the EP. Thus, taking into account the conductivity and relaxation of water, the general formula for the permittivity is as follows:

(2) $$\varepsilon \left(\omega \right)=\varepsilon_{\infty }+\frac{\sigma }{i\omega \varepsilon_{0}}+\frac{\Delta \sigma }{i\omega \varepsilon_{0}\left(1+{\left(i\omega \tau_{EP}\right)}^{-\alpha_{EP}}\right)}+\frac{\Delta \varepsilon_{water}}{1+i\omega \tau_{water}}.$$

The fitting of the dielectric spectra for the water-gas system at a gas pressure of 9 MPa (before the formation of the hydrate) using Equation (2) and the contributions of individual terms are shown in Figure 5. The red line shows the model spectrum of water at a temperature of 273 K. The spectrum of water is modeled using the Debye equation (Δε_water_ = 87.9; $\varepsilon_{\infty }$ = 5.7; τ_water_ = 17.7 × 10^−12^ s) according to the data given in the work [33]. A correct description of the EP tail increases the accuracy of the selection of the relaxation term. The procedure of fitting by Equation (2) of the dielectric spectrum in Figure 5 allows us to conclude that the EP decreases with increasing frequency, and at a frequency of ~10^4^ Hz, the EP does not contribute to the dielectric constant. Therefore, the ε′ value at a frequency of 10^5^ Hz corresponds to the static permittivity of water ε_s_.

**Figure 6:**
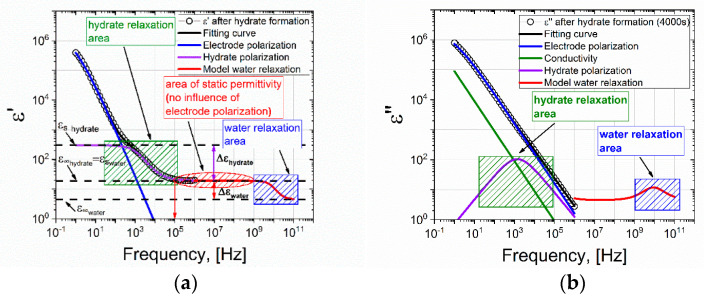
(**a**) Real and (**b**) imaginary parts of the dielectric spectrum of the water-gas system at the time t = 4000 s, after the formation of the hydrate (273 K, 9 MPa). The black line is the fitting function constructed in accordance with equation (3); the blue line is the contribution of electrode polarization; the green line represents DC conductivity; the violet line is the additional area of dispersion; and the red line is the model spectrum of water.

Now let us consider the dielectric spectrum 2 (Figure 4) after the formation of the hydrate, at the time t = 4000 s. It differs from spectrum 1 in that an additional dispersion region is observed in the frequency range 10^2^–10^4^ Hz. We assume that it is associated with the formation of a hydrate. Figure 6 shows the real and imaginary parts of the dielectric spectrum.

At the moment of hydrate formation, a part of the water in the measuring cell participates and a part remains in liquid form [34]. This is evidenced by the value of the plateau ε′ = 20 in the frequency range 10^5^–10^6^ Hz. If all the water was transformed into hydrate or turned into ice, then this value would be ε′∼4 [17]. It should be noted that the EP also does not affect the dielectric spectrum at frequencies of ~10^5^–10^6^ Hz, and the value of ε′ at a frequency of 10^5^ Hz corresponds to $\varepsilon_{\infty }$ hydrate or ε_s_ of water $\varepsilon_{\infty \;\mathrm{hydrate}}=\varepsilon_{s\;\mathrm{water}}$. To take into account the dispersion associated with the formation of the hydrate, Equation (2) is transformed into the expression

(3) $$\varepsilon \left(\omega \right)=\varepsilon_{\infty }+\frac{\sigma }{i\omega \varepsilon_{0}}+\frac{\Delta \sigma }{i\omega \varepsilon_{0}\left(1+{\left(i\omega \tau_{EP}\right)}^{-\alpha_{EP}}\right)}+\frac{\Delta \varepsilon_{water}}{1+i\omega \tau_{water}}+\frac{\Delta \varepsilon_{hydrate}}{1+{\left(i\omega \tau_{hydtate}\right)}^{\alpha_{hydrate}}},$$

where one more term is added to account for the dispersion of the hydrate.

Comparing the spectra before and after the formation of the hydrate, we see that EP is present in both cases. It strongly distorts the spectra in the region of low frequencies up to ~10^4^ Hz. However, above 10^4^ Hz, the ED has practically no effect on the dielectric dispersion. The dielectric dispersion of water is in the region of ultrahigh frequencies ~10^9^–10^11^ Hz (shown by the area with blue shading in Figure 5 and Figure 6). In our work, the experimentally accessible frequency range extends up to 10^6^ Hz. In the frequency range 10^5^–10^6^ Hz, we observe a plateau corresponding to the ε_s_ of water. Before the formation of the hydrate, ε_s_∼88, and after ε_s_∼20. As such, by observing the drop in the value of the ε_s_ of water, one can judge the formation of the hydrate. Thus, if we observe the dielectric spectrum (real part) of the water-gas system (9 MPa) versus time, the time when the value of ε′ drops at a frequency of 10^5^ Hz can be used to record the time that hydrate formation begins.

**Figure 7:**
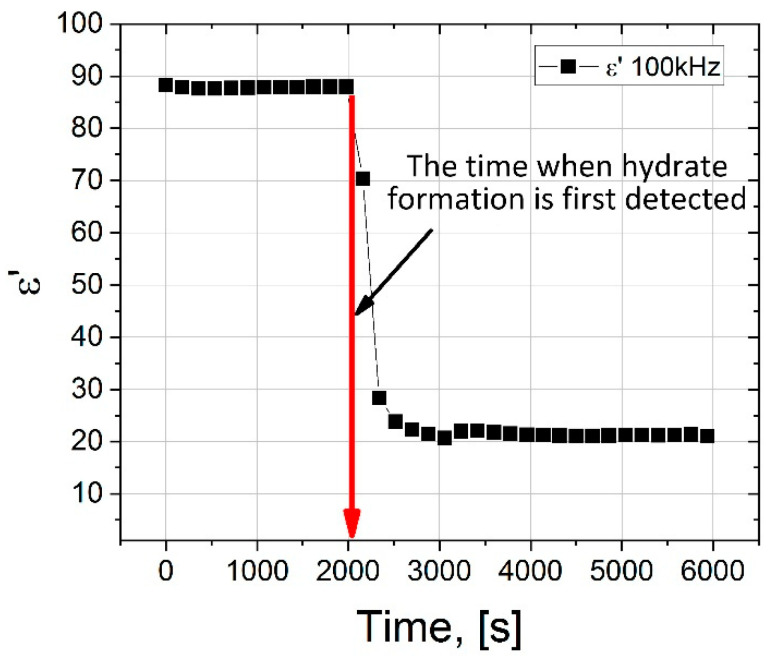
Dependence of ε′ on the time of the experiment on the formation of the hydrate (273 K, 9 MPa). The ε′ values are taken for a frequency of 10^5^ Hz.

The dependence of ε′ on the time of the experiment on the formation of the hydrate is shown in Figure 7. It can be seen that upon reaching the time t = 2000 s, a sharp decrease in the values of ε′ is observed, and in the frequency range 10^2^–10^5^ Hz in Figure 6 an additional region of dispersion appears (highlighted in green shading in Figure 6), which may be associated with the formation of a hydrate.

**Figure 8:**
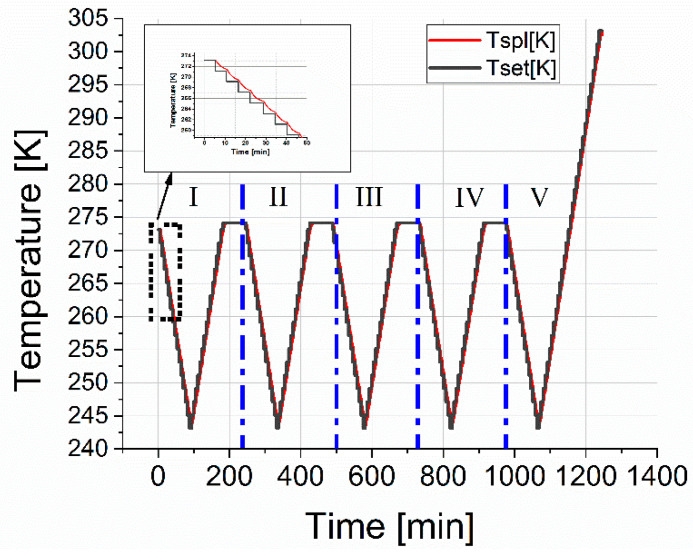
Temperature protocol for cooling and heating the measuring cell during five measurement cycles. The red line is the cell temperature; the black line is the setpoint temperatures.

The process of gas hydrate growth under static conditions (at constant temperature and pressure) takes a long time since it is associated with the diffusion restrictions of reagents into the reaction zone (free water and gas are separated by a hydrate layer) [35]. To obtain a more uniform hydrate, the already formed hydrate was subjected to freezing at 243 K and subsequent heating to a temperature of 274 K. The cell was kept at this temperature for one hour. The cycle of cooling and heating was then repeated. Five cycles were performed; in the fifth cycle, heating was carried out to a temperature of 300 K in order to estimate the decomposition temperature of the hydrate. The temperature protocol for thermocycling the cell is shown in Figure 8.

**Figure 9:**
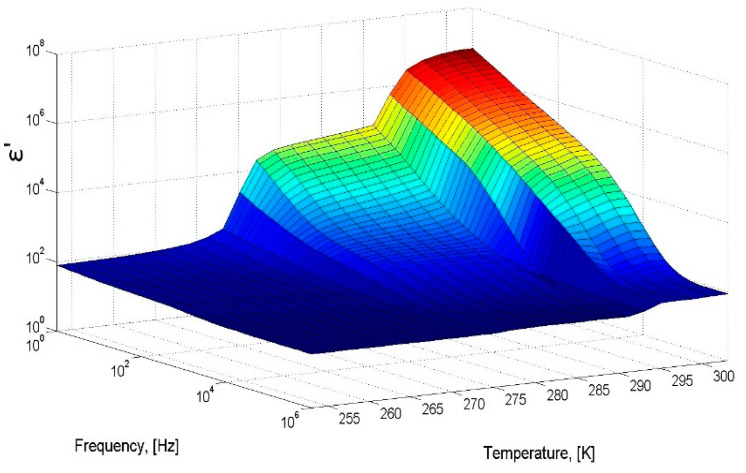
A 3-D diagram of the real part of the dielectric spectrum of the hydrate as the hydrate is heated during the fifth cycle.

Thus, within 21 h of the experiment, starting from the moment of nucleation t = 41 min, the hydrate grows in the measuring cell; however, part of the water, in this case, can remain under the hydrate layer in a free state. Cooling to 243 K leads to freezing of residual water (ice crystallization). In this case, melting should proceed in two stages. Figure 9 shows a 3-D diagram of the real part of the dielectric spectrum of the hydrate as the hydrate is heated during the fifth cycle.

**Figure 10:**
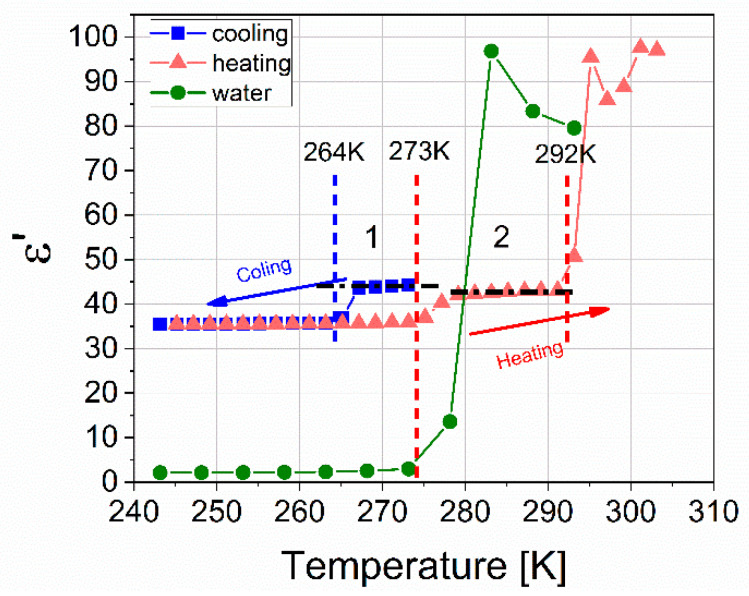
Temperature dependence of ε ‘(T) for a mixture of hydrate and ice obtained at 273 K and a pressure of 9 MPa, with a cutoff frequency of 100 kHz. Cooling of the resulting mixture in the first cycle is marked with blue symbols; the heating of the mixture in the fifth cycle is shown with red symbols. Green symbols show ice heating at atmospheric pressure.

Consider the temperature cut at a frequency of 100 kHz, shown in Figure 10. The blue symbols show the ε′(T) dependence of the formed hydrate, corresponding to cooling in the first cycle. At a temperature of 264 K, residual free water freezes, and the dielectric constant decreases from 44 to 35. Upon further cooling to 243 K, the ε′ value practically does not change and is the sum of $\varepsilon_{\infty }$ values for ice and the hydrate. The red symbols show the dependence ε′(T) of the hydrate corresponding to heating in the fifth cycle. The value of ε′(T), starting from a temperature of 243 K to 273 K does not practically change; at 273 K the contained ice melts and the value of ε′ sharply increases from 36 to 42. Further, ε′ slightly increases, and at a temperature of 291 K, a sharp increase in the amplitude to values of ≈93. This apparent increase is associated with the phenomenon of near-electrode polarization characteristic of water as temperature increases. Melting is observed in two stages: ice melts at 273 K, and hydrate decomposes at 291 K, after which the value of ε′ returns to the value of liquid water. It should be noted that the ε′ amplitude in Section 2 is somewhat lower than in Section 1 (see Figure 10), which indicates some additional hydrate formation during thermal cycling [35,36].

To assess the effect of kinetic hydrate inhibitor on the process of hydrate formation, 0.01, 0.05, and 0.1 wt. % Luvicap EG (poly (N-vinyl caprolactam) abbreviated to PVCap; 2000–8000 g/mol; BASF) aqueous solutions were used. Since the commercial product Luvicap EG is 40 wt. % solution polymer in monoethylene glycol, the tested solutions also contained 0.015, 0.075, and 0.15 wt. % of monoethylene glycol. The hydrate formation time under the same conditions as the blank tests (9 MPa and 273 K) was 800 min for a 0.01% polymer concentration and 1200 min for a 0.05% concentration (Figure S3; Supplementary). At a concentration of 0.1%, the hydrate was not formed within 48 h. An increase in the inhibitor concentration within the range investigated lead to a regular increase in the time required for the onset of gas hydrate formation, which is in good agreement with data from the literature [37,38]. Hydrate formation in the presence of an inhibitor occurs unevenly, especially at a concentration of 0.05%, where jumps and drops in the amplitude of the dielectric constant are observed. This is probably due to the involvement of inhibitor molecules in hydrogen bonding and the destruction of nucleation centers, which causes disruption to the local ordering of water molecules and prevents the formation of hydrate [39,40,41,42,43].

Nevertheless, after a certain time and corresponding temperature and pressure conditions, the hydrate is formed, but the microstructure of the hydrate with different concentrations of the inhibitor may differ from each other and, moreover, differ from the microstructure of the hydrate without the inhibitor.

Information on the structure of hydrogen bonds can be obtained from the temperature dependence of the relaxation time τ in Arrhenius coordinates, which in turn allows for the calculation of the activation energy of the dielectric relaxation process ΔE, making an assumption about the relaxation mechanism. To obtain the temperature dependence of τ and estimate the ΔE of the formed hydrates, the samples were cooled to 243 K, followed by heating to 273 K with a step of 2 °C. The complex dielectric constant of all samples ε* (ω) was approximated by a superposition of the Cole-Cole functions, taking into account electrode polarization and DC conductivity

(4) $$\varepsilon^{*}\left(\omega \right)=\varepsilon^{\prime }\left(\omega \right)-i\varepsilon^{\prime \prime }\left(\omega \right)=\varepsilon_{\infty }+\frac{\sigma }{i\omega \varepsilon_{0}}+\frac{\Delta \sigma }{i\omega \varepsilon_{0}\left(1+{\left(i\omega \tau_{EP}\right)}^{-\alpha_{EP}}\right)}+\frac{\Delta \varepsilon }{1+{\left(i\omega \tau \right)}^{\alpha }}.$$

where: ε′(ω) and ε”(ω) are the dielectric constant and dielectric loss, respectively; i is an imaginary unit; $\varepsilon_{\infty }$ is the value of the dielectric constant at high frequencies; ω represents cyclic frequency; Δε, τ, α are dielectric strength, macroscopic relaxation time and Cole-Cole parameter for the relaxation process, respectively; and ε_0_ = 8.85∙10^−12^ F/m, which is the dielectric constant of a vacuum.

**Figure 11:**
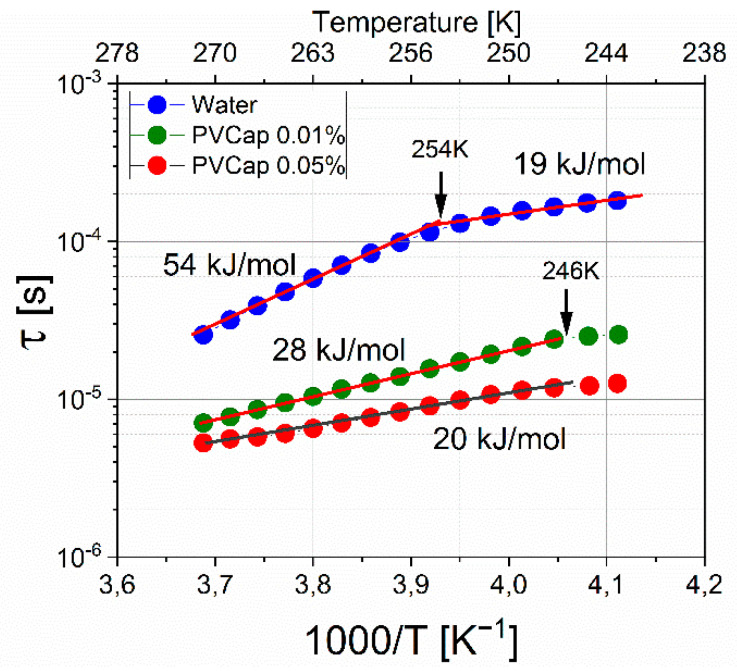
Relaxation time versus reciprocal temperature. Blue circles represent hydrate formed without the addition of an inhibitor; green circles represent hydrate formed with the addition of 0.01 wt. % PVCap; and red circles represent hydrate formed with the addition of 0.05 wt. % PVCap.

Using the results of the approximation, the dielectric relaxation times were determined. Figure 11 shows the dependences of the relaxation time τ on the reciprocal temperature 1000/T for the hydrate formed without the addition of an inhibitor and with the addition of 0.01 and 0.05 wt. % PVCap. Fitting parameters are shown in Tables S1–S3 (Supplementary). Dielectric spectroscopy is very sensitive to changes in the structure of systems with hydrogen bonds, which also include gas hydrates.

The dependences ln (τ)-(1000/T) have linear sections, which allows us to assume the activation mechanism of dielectric relaxation and, using the Arrhenius Equation (5), to estimate the activation energy ΔE of the dielectric relaxation processes:

(5) $$\tau =\tau_{0}\exp\left(\frac{\Delta E}{RT}\right),$$

where: τ is the dielectric relaxation time; τ_0_ is a constant ~10^−13^ s coinciding in order of magnitude with the period of rotational fluctuation of molecules in the equilibrium position; ΔE is the activation energy of the dielectric relaxation process; R is the gas constant; and T is the temperature.

The dependence lnτ vs (1000/T) for a hydrate formed without an inhibitor has a break; with a decrease in temperature, the activation energy changes from 54 ± 0.5 kJ/mol to 19 ± 0.5 kJ/mol. This is characteristic of ice relaxation mechanisms and indicates a change in the relaxation mechanism from orientation to ionic [17]. However, for ice I*h*, the dynamic crossover corresponds to a temperature of 232K [21,22], which indicates that the structure of the hydrate differs from ice. At 0.01% PVCap, the activation energy of the formed hydrate is 28 ± 0.5 kJ/mol, and the relaxation times are shorter in comparison with the times for the “pure” hydrate. An increase in the concentration of PVCap to 0.05% leads to even shorter relaxation times and a value of ΔE = 20 ± 0.5 kJ/mol. For the hydrate samples with PVCap at a temperature of 246 K, a small break is observed, the nature of which is not yet clear and requires additional study. The decrease in the activation energy can be explained in terms of the competition between two types of relaxation, orientational and ionic [19,20,21]. If, for some reason, the structure of the hydrogen bond network changes in the system and protons become more mobile, this leads to an increase in the number of ionic defects and an increase in the contribution of the ionic relaxation mechanism. This moves the transition point to higher temperatures. In the case of the addition of the polymer inhibitor PVCap, due to the presence of hydrophobic and hydrophilic fragments, the polymer is involved in the network of hydrogen bonds (it is disrupted), which leads to an increase in the mobility of the dipole moments of H_2_O molecules, which leads to a decrease in the activation energy and shorter relaxation times. An increase in the concentration of the inhibitor increases the contribution of these processes. The data of work [44] indicate that the effect of the inhibitor Luvicap 55W (copolymer of N-vinyl caprolactam and N-vinyl pyrrolidone) is not associated with adsorption onto nucleation sites. The data obtained on the dielectric behavior of the systems under consideration speak in favor of the involvement of the inhibitor in hydrogen bonding, which disrupts the local ordering of water molecules and prevents hydrate formation. This mechanism of action of poly (N-vinyl caprolactam) is also supported by calculations [45].

## 3. Materials and Methods

### 3.1. Materials

Deionized water (18.2 MΩ·cm at 25 °C) and poly (N-vinyl caprolactam) abbreviated to PVCap (2000–8000 g/mol; BASF, Germany) as 40 wt. % solution of polymer in monoethylene glycol were used. A synthetic natural gas was employed as a hydrate former (95.7 mol% CH_4_, 2.6 mol% C_2_H_6_, 0.8 mol% C_3_H_8_, 0.1 mol% C_4_H_10_, 0.1 mol% i-C_4_H_10_, 0.7 mol% N_2_). The hydrate-water-gas equilibrium curve is shown in Figure S2 (Supplementary).

### 3.2. Dielectric Spectroscopy

**Figure 12:**
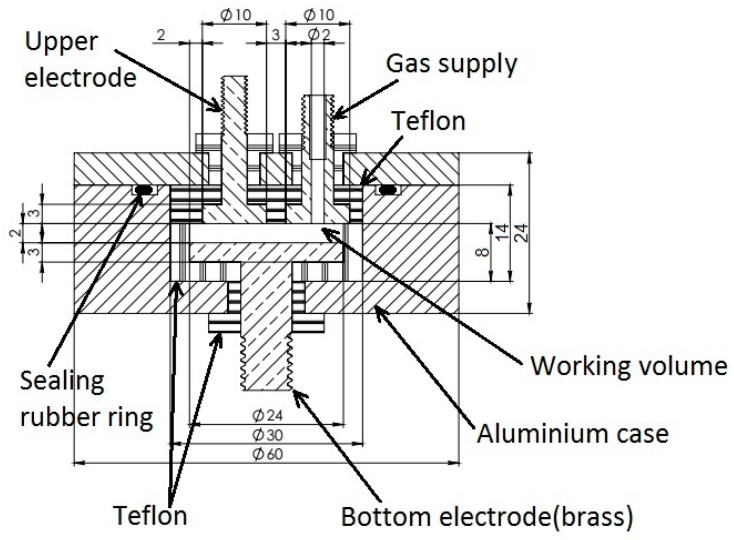
The dielectric measuring cell for studying gas hydrate formation/decomposition.

The developed dielectric cell is a 1.3 mL plane-parallel brass capacitor (Figure 12). The measuring capacitor electrodes are made of brass. The upper electrode diameter is 10 mm, the lower electrode diameter is 30 mm, the distance between the electrodes is 2 mm. The geometric capacitance of the measuring capacitor is 0.35 pF. The gas inlet is isolated from the measuring electrodes and the cell body is made of aluminum. The two body parts are tightened by six screw-bolts, and a rubber O-ring is used for sealing.

All measurements were carried out using a Novocontrol BDS-80 (NOVOCONTROL Technologies GmbH & Co. KG, Montabaur, Germany) complex. The temperature was maintained by the Quatro CryoSystem with an accuracy of ±0.1 K.

In the standard dielectric measurement experiment, 0.8 mL of deionized water was poured into the cell. The free volume of the measuring condenser (0.5 mL) was purged thrice with the chosen gas. Then, the hydrate-forming agent was fed under a pressure of 9 MPa. After that, the cell was cooled to a temperature of 273 K. From the moment the temperature reached 273 K, the dielectric spectra were measured with a step of 3 min. From a sharp decrease in the amplitude ε′ at frequencies above 10^4^ Hz, the formation of hydrate was recorded. Then the cell was kept under the given thermobaric conditions for approximately an hour to stabilize the process of hydrate formation. After the described procedures, the cell was cooled to 243 K, subsequently heated to 274 K, and held at this temperature for one hour. This was done with the aim of allowing for a more uniform distribution of the hydrate over the volume of the measuring capacitor and aiding the possible formation of additional hydrate. Five cycles of cooling and heating were carried out. During the entire experiment, a constant gas pressure of 9 MPa was maintained (see Figure 8).

### 3.3. X-ray Diffractometry

To identify the structure of gas hydrate being formed in the system, the cell was frozen at liquid nitrogen temperature and the quenched sample was taken out to be examined using powder X-ray diffraction analysis. The sample was finely ground in an aluminum mortar at the temperature of liquid nitrogen and put into a sample holder that had been pre-cooled to 173 K. A Bruker D8 Advance diffractometer (CuKα radiation (1.5418 Å)) (Bruker Corporation, Billerica, MA, USA) equipped with a TTK 450 Anton Paar low-temperature device (Anton Paar GmbH, Graz, Austria) was used. Powder diffraction pattern was recorded using 2Theta scanning mode from 3 to 45 degrees (Figure 2).

## 4. Conclusions

Understanding the structural features of gas hydrates is of great practical importance, since the technology for dealing with the negative consequences of this phenomenon depends, among other things, on the structure of the hydrate. This research expands the understanding of the properties of hydrates. As the study showed, in the water-gas hydrate system under static conditions T = 273 K and P = 9 MPa and an experiment time of 48 h, along with the formation of a hydrate, a certain amount of water remains. Nevertheless, it can be seen that the time for the onset of hydrate formation significantly increases from 800 min to 1200 min with an increase in the concentration of the inhibitor PVCap from 0.01% to 0.05%. Therefore, the DS makes it possible to observe the formation and decomposition of gas hydrates even at low concentrations in the system. The formation of hydrate affects the dielectric and relaxation characteristics. In conclusion, we can say that dielectric spectroscopy allows one to obtain important information about the processes regarding the formation of gas hydrate and the mechanism of action of inhibitors, and to study the structures of the hydrate, which are associated with relaxation time τ and the activation energy ΔE. These parameters can be used in the future to assess the effectiveness of inhibitors and promoters of hydrate formation.

## Data Availability

Data sharing is not applicable.

## Supplementary Materials

The following are available online. Figure S1: Temperature dependences ε_s_ (T) and τ (1000/T) for ice I*h*; Figure S2: Equilibrium conditions of hydrate formation for the studied gas mixture; Figure S3: Comparison of time diagrams of hydrate formation at 9 MPa versus inhibitor concentration; Table S1: Fitting parameters for water; Table S2: Fitting parameters for PVCap sample 0.01 wt. %; Table S3: Fitting parameters for PVCap sample 0.05 wt. %.

## Sample Availability

Samples of the compounds are available from the authors.
