# Supplementary material for: Advances in the Study of Gas Hydrates by Dielectric Spectroscopy
Source: Molecules. 2021 Jul 24;26(15):4459. doi: 10.3390/molecules26154459 (PMC8348377; doi:10.3390/molecules26154459)
Supplement: Supplementary file 1 [file molecules-26-04459-s001.zip › molecules-1286062-supplementary.pdf]

Advances in studies of gas hydrates by dielectric spectroscopy

Ivan Lunev [1,*], Bulat Kamaliev [1], Valery Shtyrlin [2], Yuri Gusev [1], Airat Kiiamov [3], Yulia Zaripova [4], Artur Galiullin [1], Abdolreza Farhadian[5], Mikhail Varfolomeev [4,5,*], Malcolm Kelland[6]

1 Department of Radio Electronics, Kazan Federal University, 18 Kremlevskaya street, Kazan 420008, Russian Federation; lounev75@mail.ru (I.V.); bulat1596@gmail.com (B.K.); ygusev@mail.ru (Y.G.); argj95@gmail.com (A.G.).
2 Department of Inorganic Chemistry, Kazan Federal University, 18 Kremlevskaya street, Kazan 420008, Russian Federation; valery.shtyrlin@gmail.com (V.S.).
3 Department of General Physics, Kazan Federal University, 18 Kremlevskaya street, Kazan 420008, Russian Federation; airatphd@gmail.com (A.K.).
4 Department of Physical Chemistry, Kazan Federal University, 18 Kremlevskaya street, Kazan 420008, Russian Federation; yu-ya98@yandex.ru (Y.Z.); mikhail.varfolomeev@kpfu.ru (M.V.).
5 Department of Petroleum Engineering, Kazan Federal University, 18 Kremlevskaya street, Kazan 420008, Russian Federation; AFarhadian@kpfu.ru (A.F.).
6 Department of Chemistry, Bioscience and Environmental Engineering, Faculty of Science and Technology, University of Stavanger, N-4036 Stavanger, Norway; malcolm.kelland@uis.no (M.K.).
* Correspondence: lounev75@mail.ru, mikhail.varfolomeev@kpfu.ru

Supplementary Material (SM)

Table of contents

Figure S1. Temperature dependences **(a)** ε_s (T) and **(b)** τ (1000/T) for ice I*h*. Black squares data taken from Johari et. al.[1], red empty circles experimental data measured in this work, in the developed measuring dielectric cell. The parameter ε_s is calculated according to equation 1, where $\varepsilon_s = \Delta\varepsilon + \varepsilon_\infty$.

Figure S2. Equilibrium conditions of hydrate formation for the studied gas mixture (CSMGem); equilibrium for methane hydrate is given for comparison.

Figure S3. Comparison of time diagrams of hydrate formation at 9 MPa versus inhibitor concentration. Blue symbols - distilled water; green symbols −0.01% PVCap; red symbols - 0.05% PVCap.

Figure S4. Temperature protocol for cooling and heating the measuring cell during five measurement cycles. The red line is the cell temperature, the black line is the setpoint temperatures. The inset in figure S3 shows the temperature change on an enlarged scale.

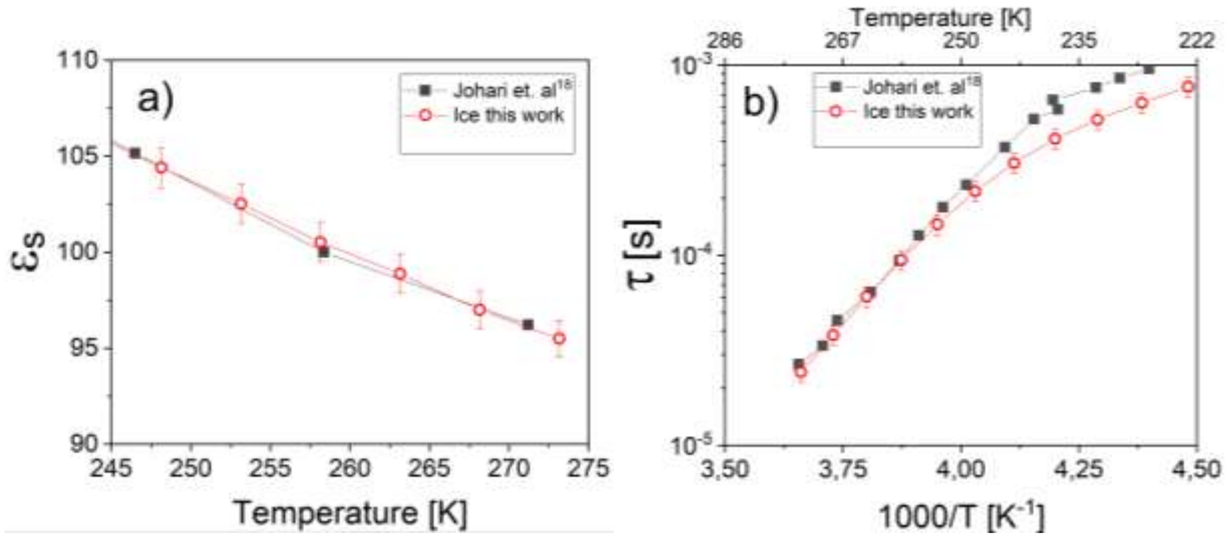

a) b)

Figure S1. Temperature dependences **(a)** ε_s (T) and **(b)** τ (1000/T) for ice I*h*. Black squares data taken from Johari et. al.[1], red empty circles experimental data measured in this work, in the developed measuring dielectric cell.

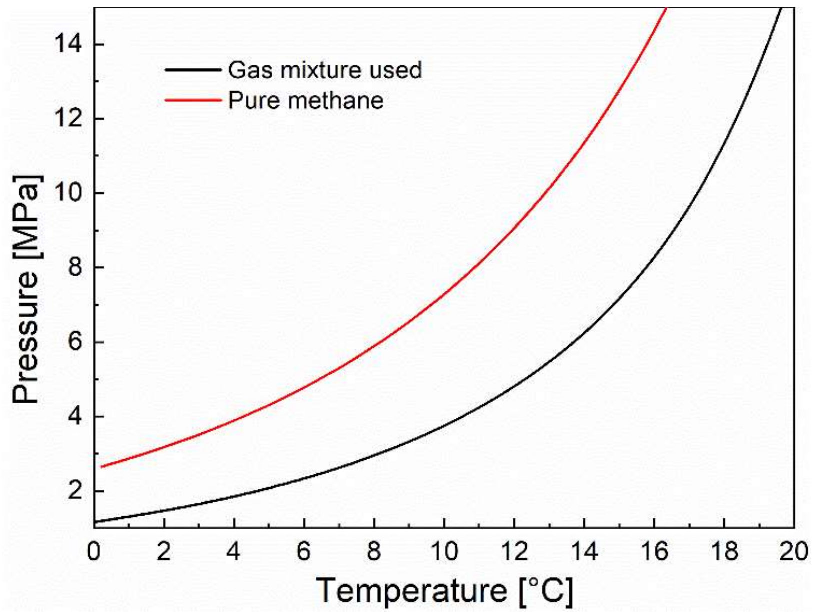

Figure S2. Equilibrium conditions of hydrate formation for the studied gas mixture (CSMGem); equilibrium for methane hydrate is given for comparison.

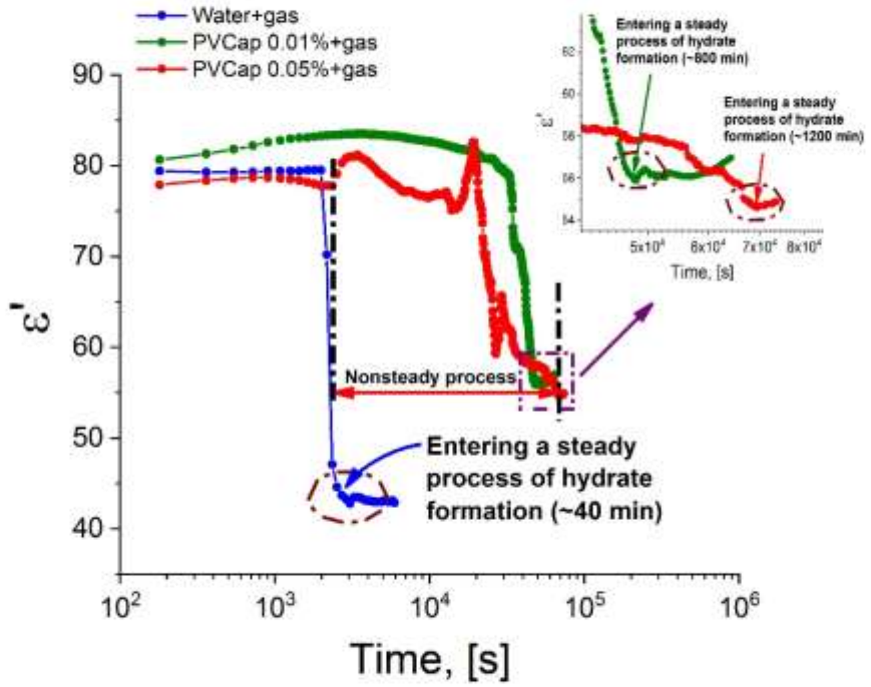

Figure S3. Comparison of time diagrams of hydrate formation at 9 MPa versus inhibitor concentration. Blue symbols - distilled water; green symbols - 0.01% PVCap; red symbols - 0.05% PVCap.

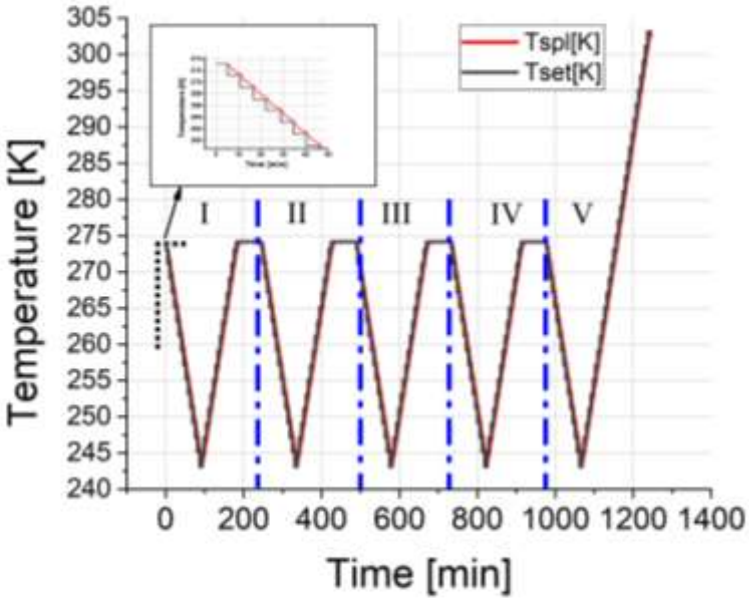

Figure S4. Temperature protocol for cooling and heating the measuring cell during five measurement cycles. The red line is the cell temperature, the black line is the setpoint temperatures.

REFERENCES

1. Johari, G.; Whalley, E. The dielectric properties of ice Ih in the range 272–133 K. *J. Chem. Phys.* **1981**, *75*, 1333-1340.
